# Comparison of small-angle neutron and X-ray scattering for studying cortical bone nanostructure

**DOI:** 10.1038/s41598-020-71190-9

**Published:** 2020-09-03

**Authors:** Elin Törnquist, Luigi Gentile, Sylvain Prévost, Ana Diaz, Ulf Olsson, Hanna Isaksson

**Affiliations:** 1 Biomedical Engineering, Lund University, PO Box 118, 221 00 Lund, Sweden; 2 Physical Chemistry, Lund University, Lund, Sweden; 3 Chemistry, University of Bari “Aldo Moro”, Bari, Italy; 4 CSGI, Sesto Fiorentino, Italy; 5 Institut Laue-Langevin, Grenoble, France; 6 Paul Scherrer Institute (PSI), Villigen, Switzerland

**Keywords:** SAXS, Bone, Characterization and analytical techniques, Imaging techniques

## Abstract

In this study, we present a combined small-angle neutron and X-ray scattering (SANS and SAXS) study of the nanoscale structure of cortical bone specimens from three different species. The variation of the scattering cross section of elements across the periodic table is very different for neutrons and X-rays. For X-rays, it is proportional to the electron density while for neutrons it varies irregularly with the atomic number. Hence, combining the two techniques on the same specimens allows for a more detailed interpretation of the scattering patterns as compared to a single-contrast experiment. The current study was performed on bovine, porcine and ovine specimens, obtained in two perpendicular directions with respect to the main axis of the bone (longitudinal and radial) in order to maximise the understanding of the nanostructural organisation. The specimens were also imaged with high resolution micro-computed tomography (micro-CT), yielding tissue mineral density and microstructural orientation as reference. We show that the SANS and SAXS patterns from the same specimen are effectively identical, suggesting that these bone specimens can be approximated as a two-component composite material. Hence, the observed small-angle scattering results mainly from the mineral-collagen contrast, apart from minor features associated with the internal collagen structure.

## Introduction

Bone is a hierarchically structured composite material. Its unique possibility to withstand mechanical loading comes from a combination of its constituents, namely type-I collagen and hydroxyapatite (HAp) crystals, and their complex structural organisation, which is built up from the molecular level to the whole organ^1–5^. To fully elucidate the contribution from each component, all length scales need to be investigated. This article focuses on the nanoscale of cortical bone where curved plate-shaped HAp crystals are interwoven in a collagen matrix, creating mineralised collagen fibrils^2,6–8^. Collagen is a triple helical molecule with diameter and length of 11 Å and 3,000 Å, respectively. The molecules congregate into fibrils where they are staggered axially such that zones of overlap and gaps form with a specific periodicity of approximately 670 Å. The mineral plates nucleate both within the periodic gap-zones and on the surface of the fibrils, and have a crystal c-axis aligned mainly along the main axis of the collagen fibril^1,3,9,10^. Water is present in different compartments: in pores and canals (on the micrometre scale), as well as loosely bound to the surfaces of collagen fibrils, and between the collagen and mineral phases, and more tightly bound to the collagen molecule and inside the mineral crystal structure^11,12^. In cortical bone, the collagen has a preferred orientation along the main axis of loading and the organisation becomes more homogeneous as the tissue matures^3,13–15^. In addition, it has been shown that cortical bone nano-structure is similar for different species^4^.

Small-angle X-ray scattering (SAXS) has been shown to be useful for investigating the nanostructure of bone as the mineral gives rise to a relatively strong scattering signal. The information obtained from SAXS studies includes average thickness, orientation, and degree of orientation of the mineral plates^4,10,14,16,17^. Neutron diffraction (ND) has previously been used to study the collagen structure, in terms of meridional and axial spacing of the collagen molecules, in both tendon and bone tissue^18–25^. Only two studies have briefly used small-angle neutron scattering (SANS) on bone^26,27^, although it is complementary to SAXS. Furthermore, no efforts have been made to compare the two techniques and clarify their complementarity.

In this study, we systematically compare small-angle neutron and X-ray scattering data from cortical bone tissue specimens from different species. The variation of the scattering cross section of elements across the periodic table is very different for neutrons and X-rays. For X-rays, it is proportional to the electron density while for neutrons it varies irregularly with the atomic number. Hence, combining SAXS and SANS allows for studying a material with two different scattering contrasts, which may give additional structural information compared to the single-contrast experiment. The current study was performed on bovine, porcine and ovine specimens, obtained in two perpendicular directions with respect to the main axis of the bone (longitudinal and radial) to maximise the understanding of the nanostructural organisation. The specimens were also imaged with high resolution micro-computed tomography (micro-CT), yielding tissue mineral density and microstructural orientation as reference.

## Materials and methods

### Specimens

**Figure 1:**
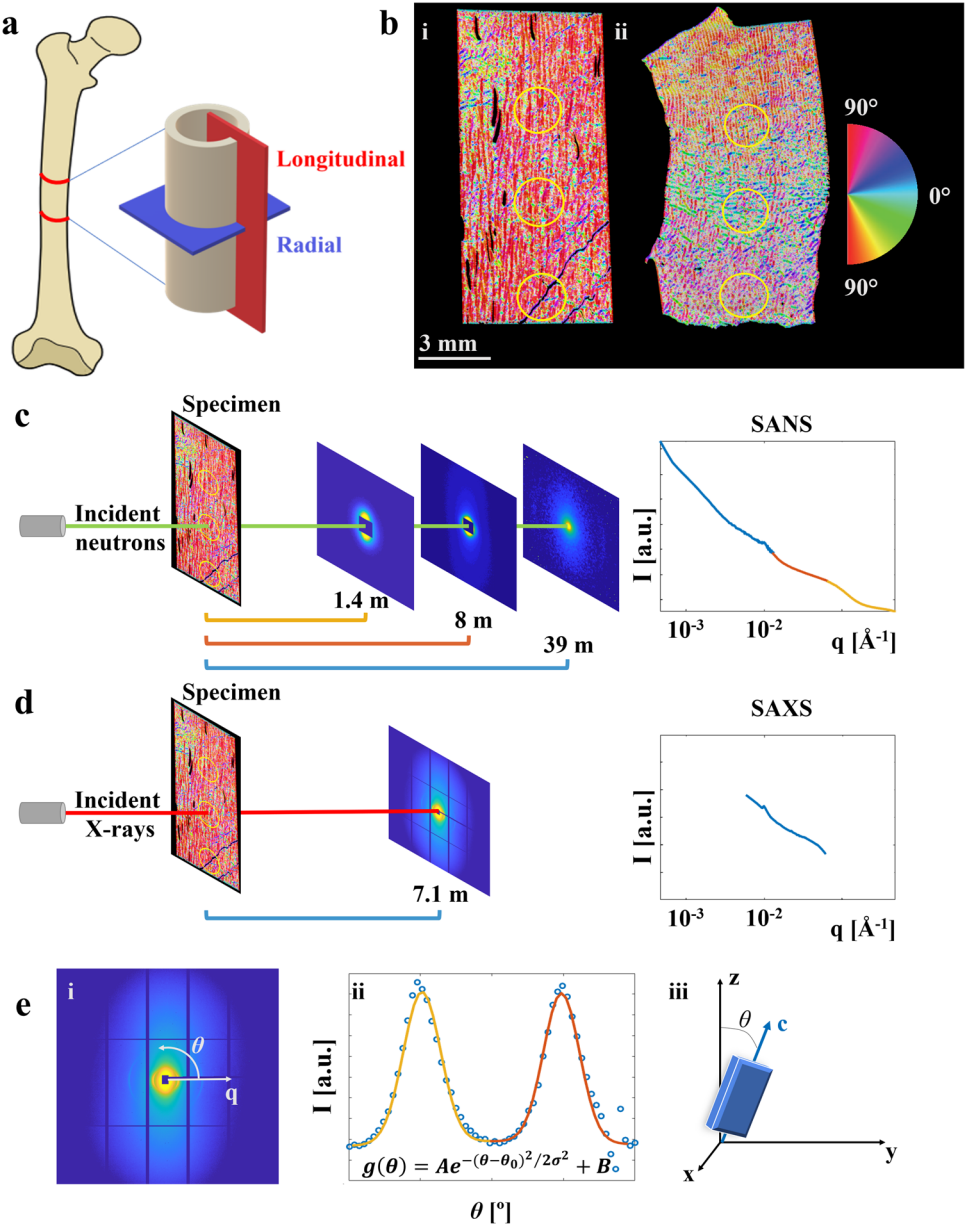
Study setup. (**a**) Visualisation of the orthogonal bone sections. The longitudinal sections were cut parallel to the main axis of the bone, and the radial sections perpendicular to it. (**b**) Microstructural orientation obtained from micro-CT images of the longitudinal (i) and radial (ii) bovine specimens. Positions for scattering measurements are indicated with yellow circles. (**c-d**) Experimental setup for SANS and SAXS, respectively, specifying distances between specimen and detector, and the resulting q-ranges (log–log scale). (**e**) Typical 2D SAXS pattern with detector edges and beamstop masked away (i), example of Gaussian fit, $g\left( \theta \right)$, to the intensity variation within the azimuthal angle from which the order parameter *S* was obtained (Eq. 4). (ii) Definition of mineral crystal c-axis orientation, *θ*, in relation to a reference direction z, both used to calculate the order parameter *S* (iii). The figure was created using Inkscape (v. 0.92), ImageJ (v1.52i), Matlab (R2019a), and GRASP Barebones (v. 8.14).

Specimens consisted of cortical bone from one bovine (29-months old) and porcine (6-months old) femora, as well as one ovine (7-months old) tibia, all obtained from the local abattoir. Each animal can be considered a young adult. Orthogonal sections in longitudinal and radial directions (Fig. 1a) with a thickness of 1 mm (0.94 ± 0.11 mm, mean ± std) were obtained from the mid-diaphysis using a diamond band saw under constant water irrigation (EXAKT 300 CP, Norderstedt, Germany). The specimens were fixed, placed in 70% ethanol, and kept refrigerated at all times except during measurements.

### Small-angle neutron scattering

*SANS* measurements were carried out at the D11 beamline, at the Institut Laue–Langevin, Grenoble, France^28^. The specimens were taken out of the 70% ethanol and the excess solvent was blotted away before placing each in separate 2 mm quartz cuvettes (100-QS, Hellma Analytics) for measurements. The longitudinal sections were placed such that the main orientation of the microstructure was aligned vertically. A wavelength of 5.6 Å in combination with specimen-to-detector distances of 39 m, 8 m and 1.4 m were utilised to acquire data in the q-range 0.00046–0.36 Å^−1^, using CERCA detectors with pixel size of 3.75 × 3.75 mm^2^ (Fig. 1c). With a beam spot size of 2 mm, scattering data was collected at three points, which were identified based on transmission measurements along the vertical midline of each specimen. An empty cuvette was measured for background reduction. After completion of the measurements, the specimens were again submerged in 70 vol% ethanol and placed back into refrigeration.

Data reduction, calibration to absolute intensity using the direct beam, and subsequent angular integration over 2π were carried out in LAMP^29^ and GRASP (GRASansP Barebones v. 8.14, Charles Dewhurst, Institut Laue Langevin, France). Data from each configuration were normalised by the intensity of the attenuated direct beam and the corresponding attenuation factor to reach absolute scale intensity. The beamstop and detector edges were masked away, and the data was integrated both over 360° (full integration), and parallel and perpendicular to the collagen fibre orientation (partial integration for anisotropy analysis). The partial integration was performed as 10 pixels wide strips around the beam centre and extending well beyond the region where counts were detected. The integrated data from the three configurations were merged using Matlab (R2019a, MathWorks Inc., MA, USA) in order to have a continuous q-range. This was done by adjusting the intensities of the mid- and high-q data ranges to overlap the low- and mid-q range data, respectively, using a factor close to 1 (± 10%).

### Small-angle X-ray scattering

*SAXS* measurements were subsequently carried out at the cSAXS beamline at PSI, Switzerland^30^. An X-ray photon energy of 12.4 keV, equivalent to a wavelength of 1 Å, was selected using a Si (111) monochromator. The beam size at the specimen position was 150 × 125 µm^2^ (horizontal × vertical), for which the beam was slightly focused using the beamline optics. The specimens were mounted directly on the specimen stage and hence were measured in air. A specimen-to-detector distance of 7.1 m and exposure time of 100 ms were utilised to acquire data in the q-range of 0.0048–0.109 Å^−1^, using a Pilatus 2 M detector with pixel size 172 × 172 µm^2^^31^ (Fig. 1d). A grid scan of 8 × 9 points was performed to cover an area of 1.2 × 1.125 mm^2^ centred at the same positions as those measured previously with SANS. The 2D scattering data was averaged to simulate the volume measured with SANS. Data reduction, calibration to absolute intensity using a glassy carbon standard^32^, and subsequent angular integration over 2π were done using in-house Matlab code (R2019a, MathWorks Inc., MA, USA)^14,16^, as well as the cSAXS Matlab base package^33^. The beamstop, edges of the detector modules, and bad pixels were masked away, and the data was integrated both over 360° (full integration), and parallel and perpendicular to the collagen fibre orientation (partial integration for anisotropy analysis). The partial integration was realised as 30 pixels wide strips around the beam centre and extending well beyond the region where counts were detected.

### Micro-computed tomography

*Micro-CT* measurements were carried out at the 4D Imaging Lab, Division of Solid Mechanics, Lund University, Lund, using a 3D X-ray microscope (Zeiss Xradia XRM520), in order to determine tissue mineral density, porosity, and orientation of the microstructures. The specimens were stacked and wrapped in saline soaked gauze during imaging. Two HAp phantoms (0.75 g/cm^3^ and 0.25 g/cm^3^, respectively) were imaged to calibrate the tissue mineral density of the specimens. A tube voltage of 80 kV and power of 70 W was used to collect 2,501 projections over 360°, each with 5 s exposure. The field-of-view (FOV) was approximately 18 × 18 mm, and the isotropic voxel size in the reconstructed images was 9.25 µm.

The micro-CT images were analysed using ImageJ v1.52i^34^. For each specimen, the three positions measured with SANS and SAXS were identified based on known distances obtained from the transmission measurements from the scattering experiments, and specific structural and compositional parameters were calculated after thresholding in the grey scale range of 140–255 (8-bit images), representing the tissue fraction above 1.0 g/cm^3^ of mineralised bone. The *minimum intensity projection* was used to visualize the micro-porosity for analysis of the microstructural orientation, yielding a specimen specific predominant orientation (Fig. 1b), using *OrientationJ*^35^. Tissue mineral density was evaluated by comparing the mean grey value to that of the phantoms and extrapolating from their known mineral density. The porosity was calculated using *BoneJ*^36^.

### Comparison of scattering data

The integrated scattering intensities (360°) from SANS and SAXS were compared in the overlapping q-range (0.0048–0.109 Å^−1^), using the common definition of q as $q = 4\pi \cdot \sin \left( {\theta /2} \right)/\lambda$, where $\theta$ is the angle between the direct and scattered beam. The intensities were adjusted by a proportionality constant, specific to the specimen and measurement position, to overlap at low q. The difference at each q, defined as the interval between two sequential SANS data points (Δq = 9.7e−4 ± 8.4e−4 Å^−1^, mean ± std), was calculated to find q-range dependent differences between the techniques, as per

1 $$ \delta I\left( q \right) = \left( {I_{X} \left( q \right) - I_{N} \left( q \right)} \right)/\left( {I_{X} \left( q \right) + I_{N} \left( q \right)} \right) $$

with ${\text{I}}_{{\text{X}}} \left( q \right)$ and ${\text{I}}_{{\text{N}}} \left( q \right)$ being the scattering intensity at a specific $q$ for SAXS and SANS, respectively. Also, the difference for the entire overlapping q-range was investigated, as per

2 $$ \delta I_{full} = \left( {\sqrt {\left( {I_{X} - I_{N} )} \right)} } \right)/\left( {\sqrt {\sum \left( {I_{N} } \right)^{2} } } \right) $$

with ${\text{I}}_{{\text{X}}}$ and ${\text{I}}_{{\text{N}}}$ being the scattering intensity for the entire q-range. Equations (1) and (2) were also used to explore the anisotropy using the partial integration (parallel and perpendicular to the collagen fibre orientation). For this the overlapping q-range was slightly smaller (0.0048–0.088 Å^−1^) due to the reduced signal-to-noise ratio at high q. In addition, integration as a function of azimuthal angle covering four partial q-ranges (0.005–0.008 Å^−1^, 0.020–0.027 Å^−1^, 0.060–0.067 Å^−1^, and 0.90–0.96 Å^−1^) was used to elucidate possible differences in anisotropy as a function of q. The partial q-ranges were chosen in between collagen peaks to avoid interference from the collagen-specific scattering (Fig. 3a,b,d). The anisotropic scattering patterns were analysed in terms of an order parameter, *S*, quantifying the orientational order of the mineral crystal plates, assuming uniaxial symmetry. Here, *S* is defined as the second order Legendre polynomial, as per

3 $$ S = \frac{1}{2}\langle3\cos^{2} \theta - 1\rangle $$

where $\theta$ is the angular deviation from the average orientation, and <  > refers to an ensemble average. The definition of $\theta$ is illustrated in Fig. 1e. Azimuthal plots $I\left( \theta \right)$ for a given q-band typically showed two peaks, that were fitted with Gaussian functions (Fig. 1e), obtaining a standard deviation $\sigma$, describing the width of the peaks. From $\sigma$, the order parameter was calculated, as per

4 $$ S = \frac{3}{2}\frac{{\mathop \smallint \nolimits_{0}^{\pi } \exp \left\{ { - (\theta - \theta_{0} )^{2} /2\sigma^{2} } \right\}\sin \theta \cos^{2} \theta d\theta }}{{\mathop \smallint \nolimits_{0}^{\pi } \exp \left\{ { - \left( {\theta - \theta_{0} } \right)^{2} /2\sigma^{2} } \right\}\sin \theta d\theta }} - \frac{1}{2} $$

### Scattering contrast

The scattering length densities of collagen and mineral were calculated using the NIST Neutron activation and scattering calculator (https://www.ncnr.nist.gov/resources/activation/), assuming chemical formulae and mass densities presented in Table 1. The partial carbonation of the bone mineral (ca. 5 wt% CO_3_^2−^ substitution^37–40^) has only a negligible effect on the scattering length densities and was hence omitted from the calculations.

**Table 1:** Chemical formulas and mass densities for type-I collagen, hydroxyapatite (HAp), and water, together with respective calculated neutron and X-ray scattering length densities.

	Formula	Mass density (g/cm^3^)	Neutron scattering length density (cm^−2^)	X-ray scattering length density (cm^−2^)
Collagen	C_75_ H_91_ N_19_ O_16_^a^	1.42^b^	2.4 × 10^10^	12.8 × 10^10^
HAp	Ca_5_(PO_4_)_3_OH^c^	3.2^d^	4.2 × 10^10^	27.3 × 10^10^
Water	H_2_O	1.0	− 0.6 × 10^10^	9.4 × 10^10^

^a^From Ref.^41^.

^b^From Ref.^42^.

^c^From Ref.^43^.

^d^From Ref.^44^.

## Results

Striking similarities between SANS and SAXS data were apparent for all measurements. Comparing the fully integrated (360°) scattering intensities in the overlapping q-region showed very small differences (≤ 15.4%) for all specimens (Table 2), with a proportionality constant of 35.9 ± 17.5 (mean ± std) for overlap of low q data in the overlapping q-range.

**Table 2:** Differences in intensity in the overlapping q-range (Eq. 2) for full (360°) and partial integration (columns three and five, respectively). The partial integration was realised parallel and perpendicular to the collagen fibre orientation, focusing on the collagen and mineral component, respectively. Intensity differences are presented as mean ± std of the three measurements per specimen.

Species	Microstructural orientation	Full integration *δI*_*full*_ (%)	In reference to the collagen fibre orientation	Partial integration *δI*_*full*_ (%)
Bovine	Longitudinal	7.1 ± 0.6	Perpendicular	9.7 ± 2.6
			Parallel	26.6 ± 7.7
	Radial	5.2 ± 2.1	Perpendicular	12.1 ± 1.9
			Parallel	15.4 ± 2.4
Porcine	Longitudinal	8.0 ± 1.3	Perpendicular	13.6 ± 3.1
			Parallel	25.4 ± 6.8
	Radial	6.6 ± 0.9	Perpendicular	14.7 ± 1.3
			Parallel	19.1 ± 6.2
Ovine	Longitudinal	15.4 ± 1.4	Perpendicular	12.0 ± 2.2
			Parallel	75.0 ± 10.1
	Radial	6.6 ± 0.0	Perpendicular	11.9 ± 1.9
			Parallel	13.5 ± 3.7

**Figure 2:**
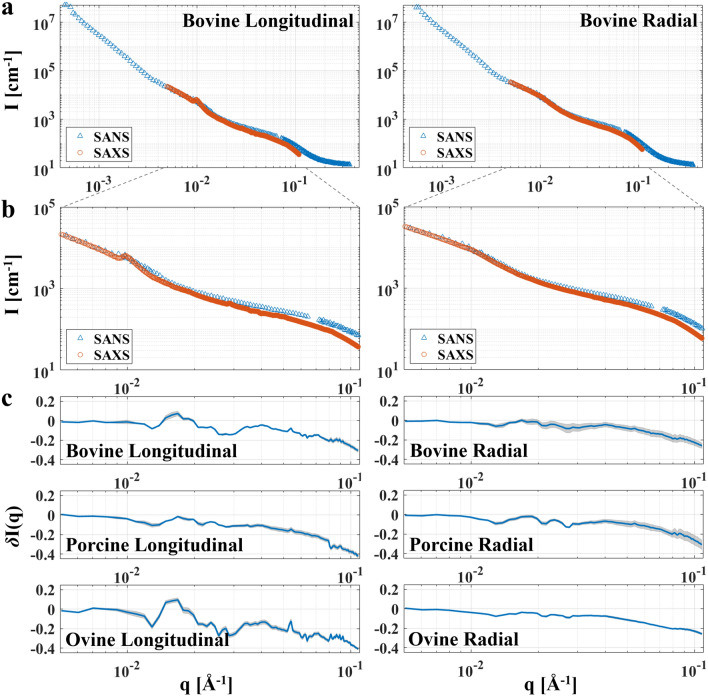
Comparison of absolute intensity for SANS and SAXS in the overlapping q-range. (**a**) Representative full 2π integration (360°, log–log scale) of SANS (∆) and SAXS (o) data from one measurement position on the longitudinal and radial bovine specimens. The SAXS data was offset to overlap with the SANS data at low q. (**b**) Zoom-in on the overlapping q-range for the plots in (**a**). (**c**) q-dependent intensity differences (linear-log scale) for full integration of SANS and SAXS scattering intensities in the overlapping q-region shown as mean (blue line) and standard deviation (shaded grey area) for the three measurement positions on each specimen. The SAXS and SANS scattering curves for the porcine and ovine specimens are available in Supplementary Figs. 2–3. The figure was created using Matlab (R2019a).

For all measurements, the difference between the techniques increased slightly as a function of q, with the SAXS intensity at higher q being lower compared to the SANS intensity (Fig. 2). Data from all specimens are available in Supplementary Figs. 1–3. For the partial integration parallel to the collagen fibre orientation, the strong collagen peaks in the SAXS data, especially from the longitudinal ovine specimen, gave rise to large differences in intensity for the longitudinal specimens (Supplementary Fig. 4). However, integration perpendicular to the collagen fibre orientation for the longitudinal specimens, and both parallel and perpendicular to the collagen fibre orientation for the radial specimens, showed the same variation (differences increased as a function of q) as the fully integrated data (Supplementary Figs. 1–5).

**Figure 3:**
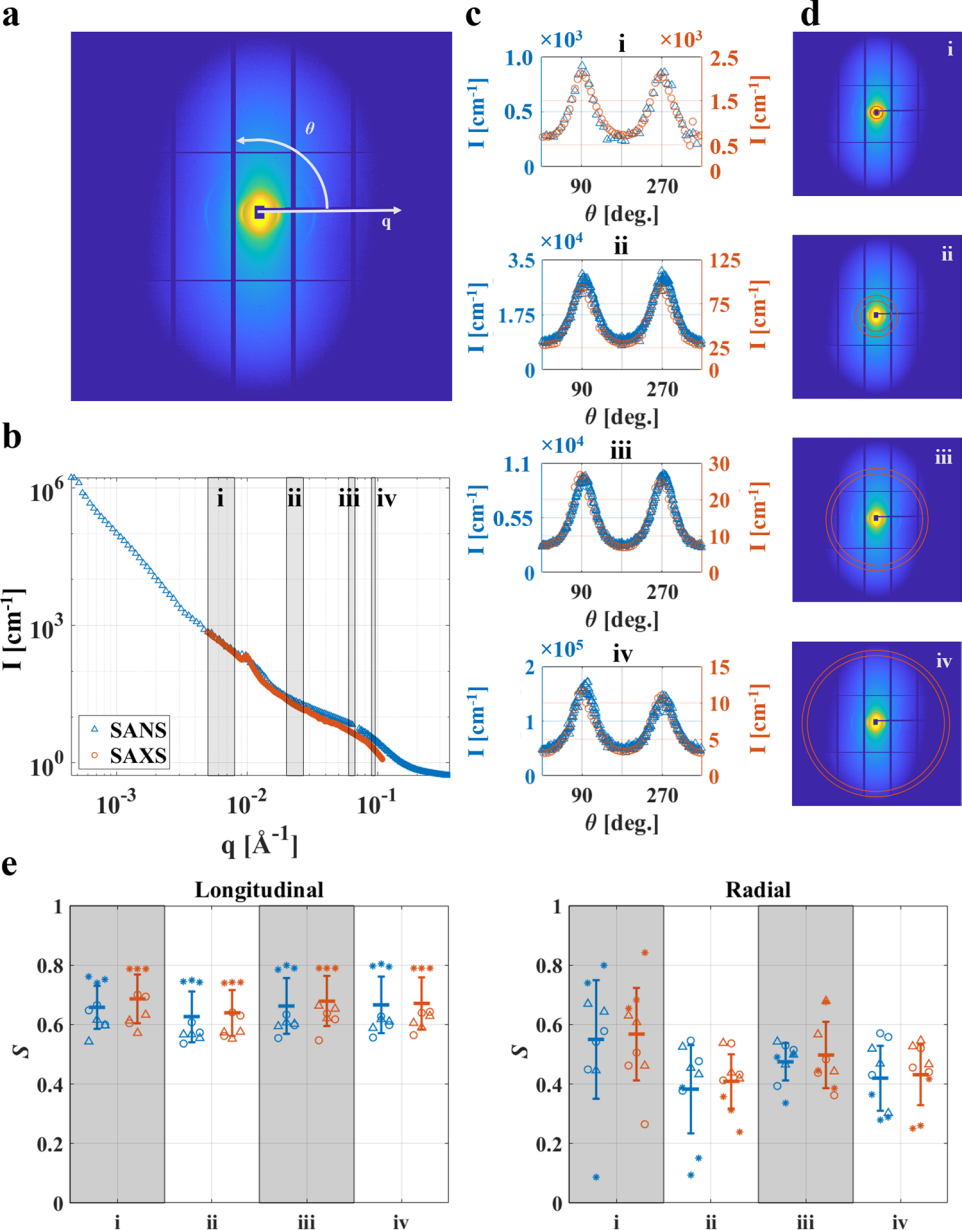
Anisotropy analysis. (**a**) Representative 2D SAXS scattering pattern from the longitudinal bovine specimen. (**b**) Integrated (360°) SANS (∆) and SAXS (o) data with the q-ranges used for azimuthal integration indicated in grey. The SAXS data was offset to overlap with the SANS data at low q. (**c**) Azimuthal plot for SANS (∆, left axis) and SAXS (o, right axis). (**d**) 2D SAXS scattering patterns with overlays in red indicating the q-range used for the azimuthal plot in **c**. (**e**) q-range dependent order parameter (*S*), for longitudinal and radial specimens, shown as mean (thick horizontal lines) of SANS (blue) and SAXS (red) measurements, standard deviation (bar), and value per measurement position for bovine (∆), porcine (o), and ovine (*). The figure was created using Matlab (R2019a).

The q-dependent azimuthal plot (Fig. 3) revealed that the orientational order (*S*), remained constant around 0.66 and 0.47 for both longitudinal and radial specimens in both SANS and SAXS (*S* = 0.66 ± 0.09, and *S* = 0.47 ± 0.14 (mean ± std), for longitudinal and radial specimens, respectively).

The micro-CT analysis of the microstructure indicated a homogeneous specimens set in terms of bone porosity and tissue mineral density (Table 3). The orientational analysis (Fig. 1b and Supplementary Fig. 9) showed a predominant orientation parallel to the long axis of the bone for the longitudinal sections (low standard deviation), and more disperse orientation for the radial sections.

**Table 3:** Micro-CT analysis. The predominant orientation was taken as the mean orientation defined in Fig. 1b (90° being along the main axis of the bone). The values presented are mean ± std of the three measurement positions on each specimen.

Species	Section	Predominant orientation (deg.)	Porosity (%)	Tissue mineral density (g/cm^3^)
Bovine	Longitudinal	90.6 ± 1.5	4.1 ± 1.2	1.35 ± 0.01
	Radial	65.6 ± 56.7	2.0 ± 0.6	1.34 ± 0.01
Porcine	Longitudinal	52.1 ± 4.1	10.7 ± 0.8	1.23 ± 0.01
	Radial	77.5 ± 38.9	4.7 ± 2.0	1.25 ± 0.02
Ovine	Longitudinal	88.1 ± 2.3	6.3 ± 1.0	1.25 ± 0.01
	Radial	105.1 ± 71.3	2.8 ± 0.6	1.27 ± 0.02

## Discussion

The purpose of this study was to compare small-angle neutron and X-ray scattering from cortical bone for a systematic analysis of the possible complementarity of the two techniques. Comparing SANS and SAXS patterns from the same specimen allows for a detailed interpretation of the scattering and the specimen nanostructure. We have found that the nanostructure of cortical bone gives rise to the same small-angle scattering pattern when using neutrons as when using X-rays. Both in terms of q- and angular dependence, the data showed striking similarities for all specimens (Fig. 2 and Supplementary Figs. 1–3). This strongly points to a two-component system at the nanoscale, i.e. a matrix that consists mainly of type-I collagen as organic component, and hydroxyapatite (HAp) as inorganic component.

The main difference in scattering intensity between the SANS and SAXS data was seen at high q, where the SAXS intensity drops two orders of magnitude following the plateau at ~ 0.08 Å^−1^ (Fig. 2a–b and Supplementary Figs. 1–3a,b), after which internal structures of the mineral crystals give rise to diffraction peaks^4^. It is worth noting that the SAXS data was offset, with the specimen and measurement specific proportionality constant calculated based on the scattering contrast, to overlap with the SANS data at low q. A Guinier region is seen in both SANS and SAXS data at ~ 0.07 Å^−1^ (Fig. 2 and Supplementary Figs. 1–3). This region yields information about the radius of gyration, corresponding to the average size of the mineral platelets in bone. Curve-fitting has previously been employed to obtain the thickness of the mineral platelets^4,14,16^. This was not done in the current study, where it was instead chosen to focus on the full overlapping q-range comparison of SANS and SAXS data. For neutrons, there is a contribution of incoherent scattering to the data, mainly due to hydrogen, which results in a constant offset. Subtracting this offset from the SANS data is expected to increase the similarities to the corresponding SAXS data. However, there is no simple way to assess this constant offset since no measurements of isotopic composition were done. In addition, the data is shown in log–log scale, meaning the subtraction needs to be very precise. Therefore, no correction for incoherent scattering was done for the SANS data. At high q, probing shorter length scales, the scattering results from the internal structure of the mineral platelets and possibly also the collagen fibres. Here, a difference between SANS and SAXS intensities is expected as it rather reflects the relative differences in atomic cross sections, which are different for SANS and SAXS. This difference at high q is further substantiated by the findings of previous neutron and X-ray diffraction studies of mineralised tissues, where the techniques have been used specifically to focus on the different constituents (mineral and collagen) at high q^18–25^. The ovine specimens (both longitudinal and radial) had slightly higher values for the intensity difference evaluated over the full q-range (Eq. 2) than the other specimens (Table 2). The q-dependent difference (Eq. 1) showed that the intensities started deviating at lower q for the radial ovine specimen in comparison to the others (Fig. 2c), which explained the higher overall difference. For the longitudinal ovine specimen, clear collagen peaks in the SAXS data reduced the similarity with the SANS data. Different from the other specimens, the radial bovine and porcine specimens, both displayed larger variations (std) in intensity differences at higher q (Fig. 2c). Comparing the scattering curves for each individual measurement position showed that, for all but the radial bovine and radial porcine specimens, the scattering curves from both SANS and SAXS were overlapping almost perfectly. For the radial bovine specimen, the three measurement positions had slightly varying SAXS scattering amplitudes. For the porcine specimen, the SAXS intensity amplitude was slightly lower for one measurement point than the others. These discrepancies resulted in more varying intensity differences between the SANS and SAXS data for these two specimens than for the others. However, for all specimens and measurements the data from each of the three measurement positions matched closely. The normalised root-mean-square-errors (NRMSEs) were 2.8 ± 2.7% and 1.5 ± 1.7% (mean ± std) for SAXS and SANS measurements, respectively. This indicates a homogeneous nanostructure throughout each specimen.

The strong similarities between the SANS and SAXS patterns indicate that the bone structure at the nanoscale can be seen as a two-component composite, the only effective contrast being between the two main constituents, namely the collagen and the HAp crystals. The collagen matrix makes up the continuous phase, with some additional internal structures, in which the mineral platelets are dispersed. The mechanical properties are greatly affected by the presence of water on all hierarchical length scales^12^, but our findings indicate that the contribution from water on the scattering is minor. The water that would yield a scattering signal in the probed q-range is that which is loosely bound to the collagen fibrils and mineral platelets. The free water in pores and canals are at a different length scale than what is probed in this study^5^, and the tightly bound water between collagen molecules and inside the mineral crystals is included as part of the collagen and mineral phase, respectively. Bound water makes up 4–4.5 wt% of cortical bone^12^, and hence the fraction of loosely bound water is even less. The specimens were stored in 70% ethanol and some of the loosely bound water could hence have been exchanged. However, the degree of exchange should be minor since the specimens were not dried prior to submersion in the solvent. The lack of significant contribution from water/solvent on the scattering data indicates that the amount of loosely bound water in cortical bone is minor when it comes to nano-structural characterisation.

Since, for a binary system, the scattering intensity $I\left( q \right)$ can be interpreted as

5 $$ I\left( q \right)\sim \Delta \rho^{2} S_{eff} \left( q \right)\langle{P\left( q \right)}\rangle $$

where

6 $$ \Delta \rho = \left( {\rho_{m} - \rho_{c} } \right) $$

is the contrast between the mineral and collagen, $\rho_{m}$ and $\rho_{c}$, being the mineral and collagen scattering length densities, respectively. $\langle{P\left( q \right)}\rangle$ is the average form factor of the mineral platelets (averaged over e.g. a polydisperse size/thickness distribution), and $S_{eff} \left( q \right)$ is an effective structure factor, reporting on the distribution of the mineral platelets over the collagen matrix. At low-q, the scattering pattern shows a characteristic strong upturn at lower *q*-values. This upturn is associated with $S_{eff} \left( q \right)$ and implies that the mineral platelets are not distributed homogeneously, but heterogeneously across the collagen matrix, with domains of higher and lower mineral concentrations. As the mineral platelets are oriented parallel to the collagen fibres, they give rise to an anisotropic scattering pattern with dominating scattering perpendicular to the collagen fibres. Thus, $S_{eff} \left( q \right)$ mainly reports on the concentration fluctuation in the plane perpendicular to the collagen fibres. We have previously^4^ described this in terms of a fractal structure factor, using an $S_{eff} \left( q \right)$ of the form $S_{eff} = \left( {1 + Aq^{ - D} } \right)$, where *D* is a mass fractal dimension and *A* is a constant related to the size and density of the mineral clusters^45^. In our previous study using SAXS we obtained *D* $\approx$ 2.3 ± 0.2 (mean ± std) when comparing different species^4^. Here, $D$ is obtained from the SANS data, which extends to lower q-values, as *D* $\approx$ 3.3 ± 0.1. The maximum dimension of a mass fractal is 3. Hence, while $S_{eff} \left( q \right)$ clearly demonstrate a heterogeneous distribution of mineral platelets, it is not clear how to interpret $S_{eff} \left( q \right)$ quantitatively.

As intensity scales with the square of the contrast, which is different for neutrons and X-rays, one can expect a factor between SANS and SAXS of 65 for a pure collagen-HAp composite, based on the scattering length densities shown in Table 1. Assuming a hydrated (10 vol.% water) collagen matrix, i.e. taking into account the loosely bound water discussed previously, reduces the proportionality factor to 50. For all specimens and measurement positions, the absolute SANS intensity was lower than the corresponding absolute SAXS intensity, and hence a proportionality constant larger than one was obtained as the SANS data was adjusted to overlap the SAXS data at low-q in the overlapping q-range. The proportionality constant was similar for all measurements except for the radial ovine specimen. The mean ± std was 28.5 ± 5.2 when excluding the radial ovine specimen, for which the proportionality constant was 72.4 ± 1.5 (mean ± std). The proportionality constant for the radial ovine specimen is a clear outlier, whose origin requires further analysis. The specimen was harvested from the same anatomical site as the longitudinal ovine specimen, and hence no significant compositional differences were expected. Looking at the specimen specific data, it was seen that the radial ovine specimen had overall higher SAXS intensities compared to all other specimens. This explains the higher proportionality constant for this specimen as compared to the others. However, why the proportionality constants of the data differ from the theoretical one is not clear.

A first order collagen peak was seen in the integrated SANS and SAXS data for all longitudinal specimens, as well as in the SAXS data for the radial bovine and porcine specimens (Supplementary Figs. 1–3). The peak location corresponded to a collagen d-spacing of ~ 640 Å, which is in agreement with previous studies on mineralised bone^18^. The peak was more defined in the SAXS data than in the SANS data, most likely due to the higher instrument resolution for SAXS. The collagen signal from the longitudinal ovine specimen was stronger than for the other specimens in both SANS and SAXS measurements. The clear first order collagen peak seen in all measurements on the longitudinal ovine specimen was initially thought to be due to a lower mineral content compared to the other specimens. However, the tissue mineral density measured from the micro-CT images indicated the same amount of mineral in all specimens. Nevertheless, it is still possible that the collagen/mineral ratio was larger in the ovine specimens than in the others, possibly due to them being taken from a different anatomical location (tibia as opposed to femur). The collagen fibril structure is periodic in the axial direction and a more regular periodicity of the collagen fibrils can be assumed for the ovine specimens due to the higher intensity of the collagen peaks with respect to the bovine and porcine specimens. In fact, also higher order peaks were distinguishable in the SAXS data in the fibre direction (Supplementary Fig. 4). The partially integrated SAXS signal from the longitudinal ovine specimen again showed clearer collagen peaks than any of the other specimens, resulting in a larger overall difference between integrated SAXS and SANS intensities (Table 2). Due to the anisotropic scattering pattern, the data is smeared differently parallel and perpendicular to the fibre direction. However, this difference seems to be negligible for these specimens as the SANS and SAXS curves are very similar for both integration directions (Supplementary Figs. 4–5).

Also the azimuthal plots at different q-bands showed striking similarities between SANS and SAXS data (Fig. 3c). It revealed that the nanostructural organisation in the radial specimens, determined from both SANS and SAXS data, remained at a lower orientational order (*S* = 0.47 ± 0.14, mean ± std) compared to the longitudinal specimens, where all measurements showed a higher orientational order (*S* = 0.66 ± 0.09, mean ± std) (Fig. 3e). The spread (std = 0.09 and 0.08 for SANS and SAXS, respectively) in orientational order among the longitudinal specimens was low, indicating a homogeneous nanostructure. For the radial specimens, the lower orientational order and the larger spread (std = 0.13 and 0.12 for SANS and SAXS, respectively) showed a more heterogeneous nanostructure, as was expected and discussed elsewhere^14^, and in agreement with the orientational parameters obtained from the micro-CT analysis (Table 3). Previous studies have reported a different measurement of orientational order, *degree of orientation,* and although not directly comparable, the values of the order parameter *S* reported here are in agreement with the previously reported *degree of orientation*, for both longitudinal and radial specimens in that longitudinal sections show a higher order of organisation than radial sections^14,16^. The longitudinal ovine specimen showed higher orientational order (*S* = 0.77 ± 0.03, mean ± std) for all measurement points and q-bands as compared to the longitudinal bovine and porcine specimens (*S* = 0.59 ± 0.03 and *S* = 0.60 ± 0.04, respectively, mean ± std). However, the low standard deviations showed that the orientational order remained constant for each specimen over all investigated q-ranges. The same trend was seen for the radial specimens, with *S* = 0.49 ± 0.10, *S* = 0.50 ± 0.07, and *S* = 0.38 ± 0.23 (mean ± std) for the bovine, porcine, and ovine specimen, respectively, over all investigated q-ranges. The high std for the radial ovine specimen could at a first glance indicate the opposite, i.e. a change in orientational parameter over the investigated q-ranges. However, when looking at the order parameter for each measurement position on the specimen, it is seen that the variations are due to one measurement position on the specimen being an outlier in that it displays lower values than the other positions, especially for lower q. This could indicate inhomogeneities in the specimen or an issue with the measurement, the latter reason being more credible since the SAXS data does not show the same trend.

The orientational parameters obtained from the micro-CT measurements indicated a well aligned microstructure in the longitudinal specimens, with a predominant orientation parallel with the main axis of the bone (vertical axis of the specimen) for the bovine and ovine specimens (Table 3). For the porcine specimen, the predominant orientation angle of 52.1° indicated that the microstructure was not aligned parallel to the vertical axis of the specimen. However, the low variation of microstructural orientation in the specimen (low std of the predominant orientation) meant that the specimen could still be considered longitudinal, and the discrepancy in orientation angle is due to imperfect specimen machining rather than improper choice of anatomical location. For the radial specimens, no clear predominant orientation was seen (high std). As has been discussed previously, this lack of a clear dominant orientation comes from the concentric lamellae seen when looking at radial cross sections of cortical bone containing osteons^14,16^.

## Limitations

Due to the selection of beamstop for the high-q SANS measurements (1.4 m sample-detector distance), no overlap between mid- and high-q ranges were obtained after 2π integration of the 2D scattering, resulting in lack of data in the q-range of 0.064–0.075 Å^−1^. However, no significant features are expected in this q-range, and by comparing the data integrated over 2π to data where also the corners of the detector banks were included (“full-detector integration”), it is clear that no significant information was lost (Supplementary Figs. 6–8). Merging of the mid- and high-q ranges was done such that it matched the merged full-detector integration.

The resolution for the SAXS measurements was considerably higher than for the SANS measurements^28,30^. Nevertheless, the resolution of the SANS data (Δλ/λ = 9%) is more than enough to properly resolve any characteristic features in the scattering data. As the polydispersity of the mineral platelets is relatively high^4^, the shape of the scattering curves are due to the polydispersity of the scattering particles rather than the wavelength distribution. The lower resolution of SANS compared to SAXS further explains the intensity differences seen around the collagen peaks and thereby indicates that if the resolution was more equal, there may have been even larger similarities between the techniques.

The difference in beam dimension for SANS and SAXS was in part accounted for by performing grid scans with SAXS. Still, the volumes measured were not exactly the same for the two techniques (3.14 mm^3^ and 1.35 mm^3^ for SANS and SAXS, respectively). However, the micro-CT data shows that the micro-structure inside the measured volumes for SANS were very homogeneous. Also, both SANS and SAXS measurements covered a relatively large volume. Hence enough averaging is done for smaller structural discrepancies not to have a noticeable effect on the resulting data.

The specimens were stored in ethanol and measured in air, resulting in dehydration of the tissue from both. It is known that drying and ethanol fixation of non-mineralised tissue has an effect on the collagen^33,46^. However, due to the mineralisation of the collagen matrix in bone, changes due to dehydration are less prominent^11^. Since both SANS and SAXS measurements were carried out in the same manner, the same amount of dehydration and potential change of collagen organisation should have occurred. Thus, the data is still comparable. Furthermore, since the specimens were not dried prior to submersion in ethanol, and the fact that the solvent was 70%, we are confident that enough water remain for there to have been a possible contribution to the scattering pattern.

## Conclusions

We have shown that SANS and SAXS patterns from different sections of cortical bone from different species are essentially identical, apart from a contribution of incoherent scattering for SANS, and a proportionality factor reflecting the different in contrast between the techniques, within the studied q-range. This implies that within this q-range the bone specimens can be considered a binary composite material in the analysis of scattering data for structural characterisation, with effective contrast between the organic collagen matrix and the inorganic HAp mineral platelets. Thus, the small-angle scattering from these bone specimens can be fully understood as arising from mineral platelets distributed within a collagen fibre matrix, where the enhanced scattering at lower q-values shows that the mineral distribution is heterogeneous in the plane perpendicular to the collagen fibre axis, with mineral rich and mineral poor domains on the 1,000 Å length scale.

## Supplementary information

Supplementary information.

## References

[1] Stock SR (2015). The mineral–collagen interface in bone. Calcif. Tissue Int..

[2] Reznikov N, Bilton M, Lari L, Stevens MM, Kröger R (2018). Fractal-like hierarchical organization of bone begins at the nanoscale. Science.

[3] Burger C (2008). Lateral packing of mineral crystals in bone collagen fibrils. Biophys. J..

[4] Døvling Kaspersen J (2016). Small-angle X-ray scattering demonstrates similar nanostructure in cortical bone from young adult animals of different species. Calcif. Tissue Int..

[5] Reznikov N, Shahar R, Weiner S (2014). Bone hierarchical structure in three dimensions. Acta Biomater..

[6] Bala Y, Farlay D, Boivin G (2013). Bone mineralization: from tissue to crystal in normal and pathological contexts. Osteoporos. Int..

[7] Fratzl P, Schreiber S, Boyde A (1996). Characterization of bone mineral crystals in horse radius by small-angle X-ray scattering. Calcif. Tissue Int..

[8] Lees S (1979). A model for the distribution of HAP crystallites in bone-an hypothesis. Calcif. Tissue Int..

[9] Fratzl, P. & Paris, O. Complex biological structures: collagen and bone. In *Neutron Scattering in Biology*, 205–223 (Springer, Berlin, 2006). 10.1007/3-540-29111-3_11.

[10] Fratzl P, Fratzl-Zelman N, Klaushofer K, Vogl G, Koller K (1991). Nucleation and growth of mineral crystals in bone studied by small-angle X-ray scattering. Calcif. Tissue Int..

[11] Unal M, Yang S, Akkus O (2014). Molecular spectroscopic identification of the water compartments in bone. Bone.

[12] Granke M, Does MD, Nyman JS (2015). The role of water compartments in the material properties of cortical bone. Calcif. Tissue Int..

[13] Isaksson H (2010). Collagen and mineral deposition in rabbit cortical bone during maturation and growth: effects on tissue properties. J. Orthop. Res..

[14] Törnquist E, Isaksson H, Turunen MJ (2019). Mineralization of cortical bone during maturation and growth in rabbits. Bone Miner. Metab..

[15] Legros R, Balmain N, Bonel G (1987). Age-related changes in mineral of rat and bovine cortical bone. Calcif. Tissue Int..

[16] Turunen MJ (2016). Bone mineral crystal size and organization vary across mature rat bone cortex. J. Struct. Biol..

[17] Bünger MH (2006). Bone nanostructure near titanium and porous tantalum implants studied by scanning small angle X-ray scattering. Eur. Cells Mater..

[18] Bonar LC, Lees S, Mook HA (1985). Neutron diffraction studies of collagen in fully mineralized bone. J. Mol. Biol..

[19] Lees S, Mook HA (1986). Equatorial diffraction spacing as a function of water content in fully mineralized cow bone determined by neutron diffraction. Calcif. Tissue Int..

[20] Lees S, Barnard SM, Mook HA (1987). Neutron studies of collagen in lathyritic bone. Int. J. Biol. Macromol..

[21] Skakle JMS, Aspden RM (2002). Neutron diffraction studies of collagen in human cancellous bone. J. Appl. Crystallogr..

[22] White SW, Hulmes DJS, Miller A, Timmins PA (1977). Collagen-mineral axial relationship in calcified turkey leg tendon by X-ray and neutron diffraction. Nature.

[23] Wess TJ, Miller A, Bradshaw JP (1990). Cross-linkage sites in type I collagen fibrils studied by neutron diffraction. J. Mol. Biol..

[24] Lees S, Capel M, Hukins DWL, Mook HA (1997). Effect of sodium chloride solutions on mineralized and unmineralized turkey leg tendon. Calcif. Tissue Int..

[25] Lees S, Bonar LC, Mook HA (1984). A study of dense mineralized tissue by neutron diffraction. Int. J. Biol. Macromol..

[26] Choi Y, Shin EJ, Seong BS, Paik DJ (2012). Application of small angle neutron scattering on the analysis of Korean compact jaw bone. Met. Mater. Int..

[27] Choi Y (2018). Neutron scattering on humane compact bone. Phys. B Condens. Matter.

[28] Lindner P, Schweins R (2010). The D11 small-angle scattering instrument: a new benchmark for SANS. Neutron News.

[29] Richard D, Ferrand M, Kearley GJJ (1996). Analysis and visualisation of neutron-scattering data. J. Neutron Res..

[30] Bunk O et al. Multimodal x-ray scatter imaging. *New J. Phys.* **11**, (2009).

[31] Kraft P (2009). Performance of single-photon-counting PILATUS detector modules. J. Synchrotron Radiat..

[32] Allen AJ, Zhang F, Joseph Kline R, Guthrie WF, Ilavsky J (2017). NIST Standard reference material 3600: Absolute intensity calibration standard for small-angle X-ray scattering. J. Appl. Crystallogr..

[33] Turunen MJ, Khayyeri H, Guizar-Sicairos M, Isaksson H (2017). Effects of tissue fixation and dehydration on tendon collagen nanostructure. J. Struct. Biol..

[34] Schneider CA, Rasband WS, Eliceiri KW (2012). NIH Image to ImageJ: 25 years of image analysis. Nat. Methods.

[35] Püspöki, Z., Storath, M., Sage, D. & Unser, M. Transforms and operators for directional bioimage analysis: a survey. In *Focus on Bio-Image Informatics* 69–93 (Springer, 2016). 10.1007/978-3-319-28549-8_3 27207363

[36] Doube M (2010). BoneJ: free and extensible bone image analysis in ImageJ. Bone.

[37] Lee CA, Einhorn TA (2001). The Bone Organ System.

[38] Boskey AL, Coleman R (2010). Aging and bone. Crit. Rev. ORAL Biol. Med..

[39] Madupalli H, Pavan B, Tecklenburg MMJ (2017). Carbonate substitution in the mineral component of bone: discriminating the structural changes, simultaneously imposed by carbonate in A and B sites of apatite. J Solid State Chem..

[40] Landi E, Celotti G, Logroscino G, Tampieri A (2003). Carbonated hydroxyapatite as bone substitute. J. Eur. Ceram. Soc..

[41] Information, National Center for Biotechnology. PubChem Database. CID=6913668. https://pubchem.ncbi.nlm.nih.gov/compound/6913668.

[42] Lees S, Heeley JD (1981). Density of a sample bovine cortical bone matrix and its solid constituent in various media. Calcif. Tissue Int..

[43] Haverty D, Tofail SAM, Stanton KT, Mc Monagle JB (2005). Structure and stability of hydroxyapatite: density functional calculation and Rietveld analysis. Phys. Rev. B Condens. Matter Mater. Phys..

[44] Larrañaga MD, Lewis RJ, Lewis RA (2016). Hawley’s Condensed Chemical Dictionary.

[45] Teixeira J (1988). Small-angle scattering by fractal systems. J. Appl. Crystallogr..

[46] Masic A (2015). Osmotic pressure induced tensile forces in tendon collagen. Nat. Commun..

