# Supplementary material for: Comparison of small-angle neutron and X-ray scattering for studying cortical bone nanostructure
Source: Sci Rep. 2020 Sep 3;10:14552. doi: 10.1038/s41598-020-71190-9 (PMC7471706; doi:10.1038/s41598-020-71190-9)
Supplement: Supplementary file 1 — Supplementary information. [file 41598_2020_71190_MOESM1_ESM.docx]

**Supplementary material:**

**Comparison of small-angle neutron and X-ray scattering for studying cortical bone nanostructure**

Elin Törnquist, Luigi Gentile, Sylvain Prévost, Ana Diaz, Ulf Olsson, Hanna Isaksson

<https://doi.org/10.1038/s41598-020-71190-9>


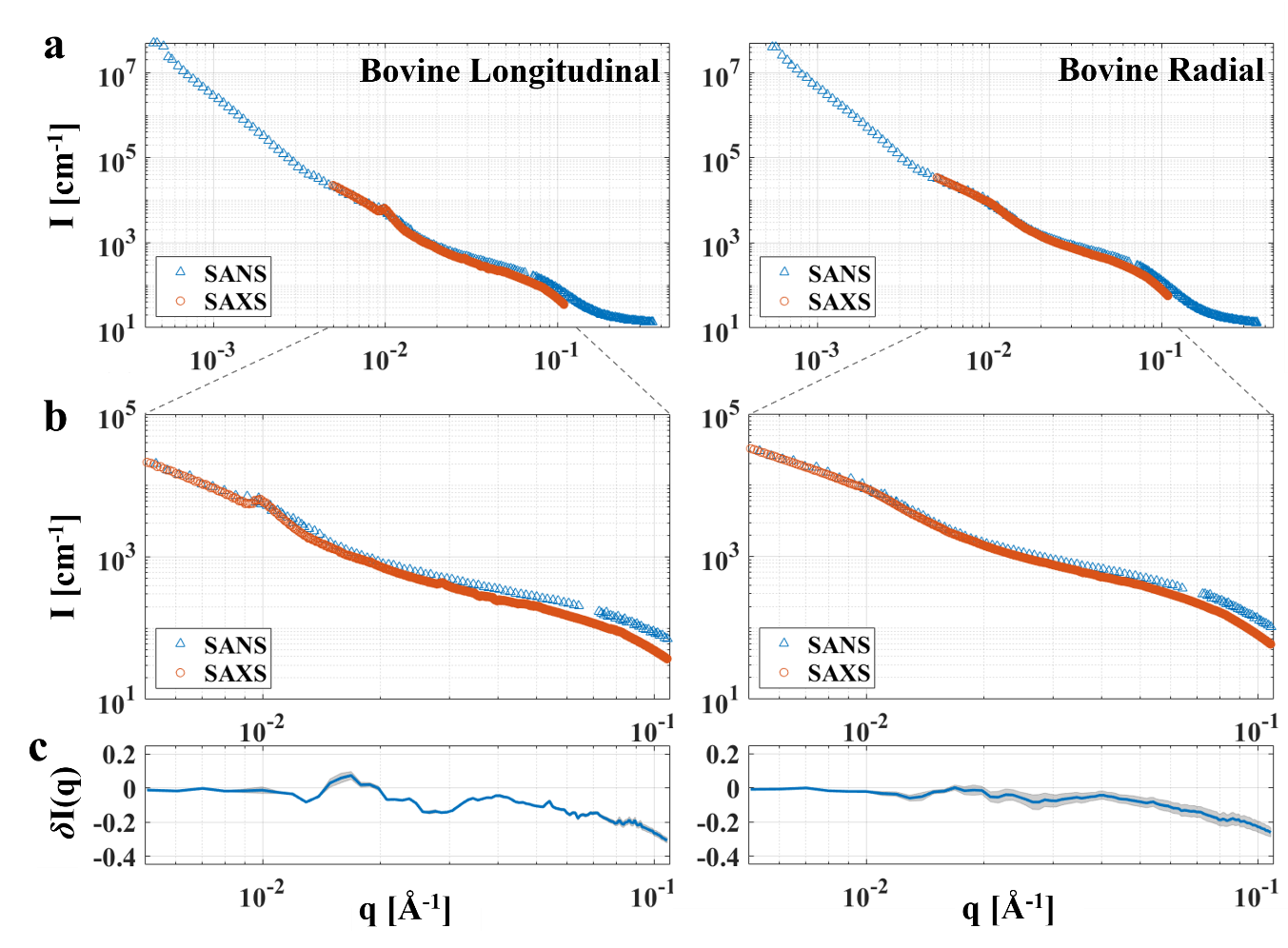


**Supplementary Figure 1**. **a**) Full 2π integration (360°, log-log scale) of SANS (∆) and SAXS (o) patterns for the **bovine** longitudinal (left) and radial (right) specimens. The SAXS data was offset to overlap with the SANS data at low q. **b**) Zoom-in on the overlapping q-range for the plots in **a**). **c**) q-dependent intensity differences (linear-log scale), shown as mean (blue line) and standard deviation (grey shaded area) for the three measurement points. The figure was created using Matlab (R2019a).


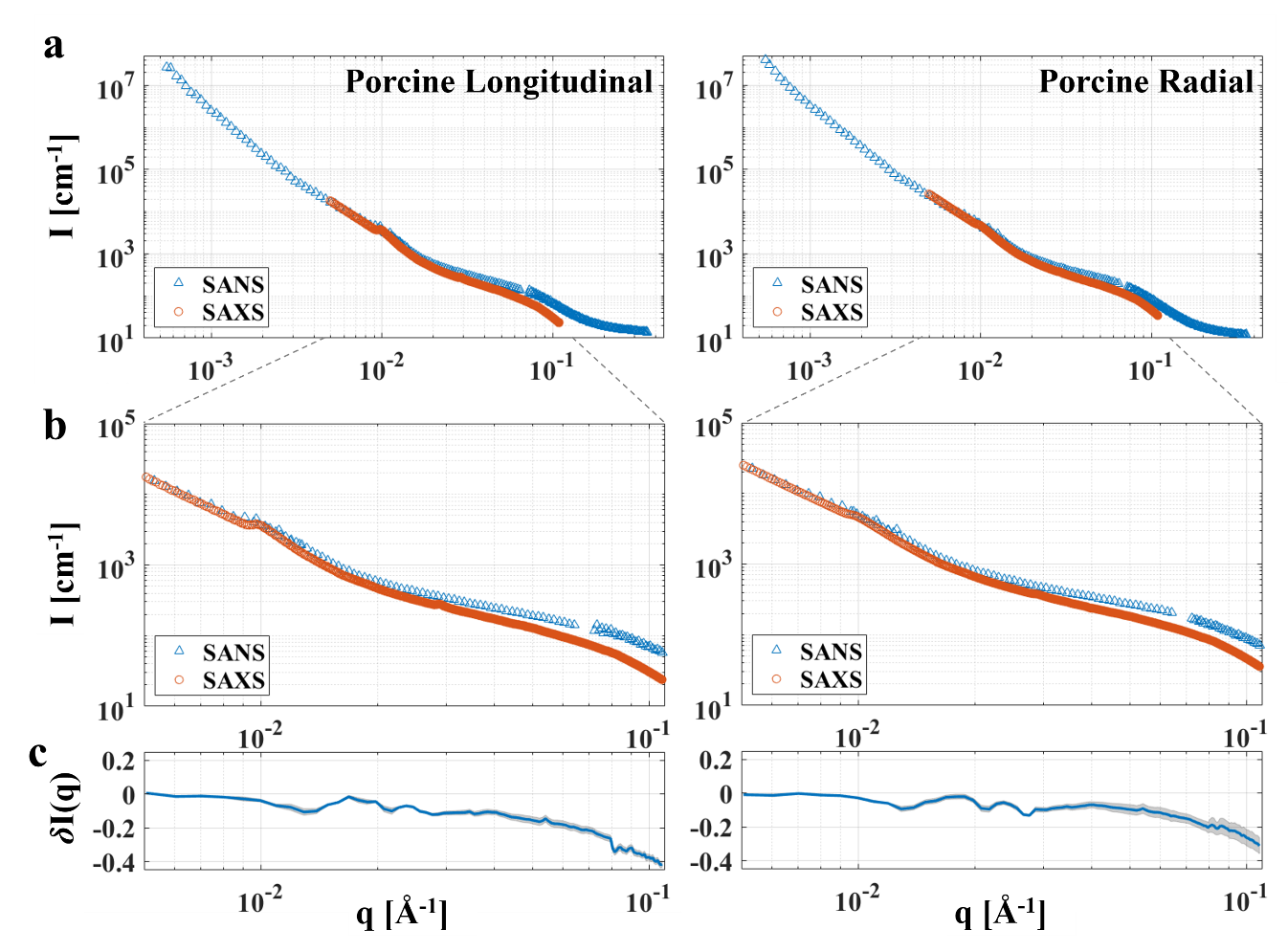


**Supplementary Figure 2. a**) Full 2π integration (360°, log-log scale) of SANS (∆) and SAXS (o) patterns for the **porcine** longitudinal (left) and radial (right) specimens. The SAXS data was offset to overlap with the SANS data at low q. **b**) Zoom-in on the overlapping q-range for the plots in **a**). **c**) q-dependent intensity differences (linear-log scale), shown as mean (blue line) and standard deviation (grey shaded area) for the three measurement points. The figure was created using Matlab (R2019a).


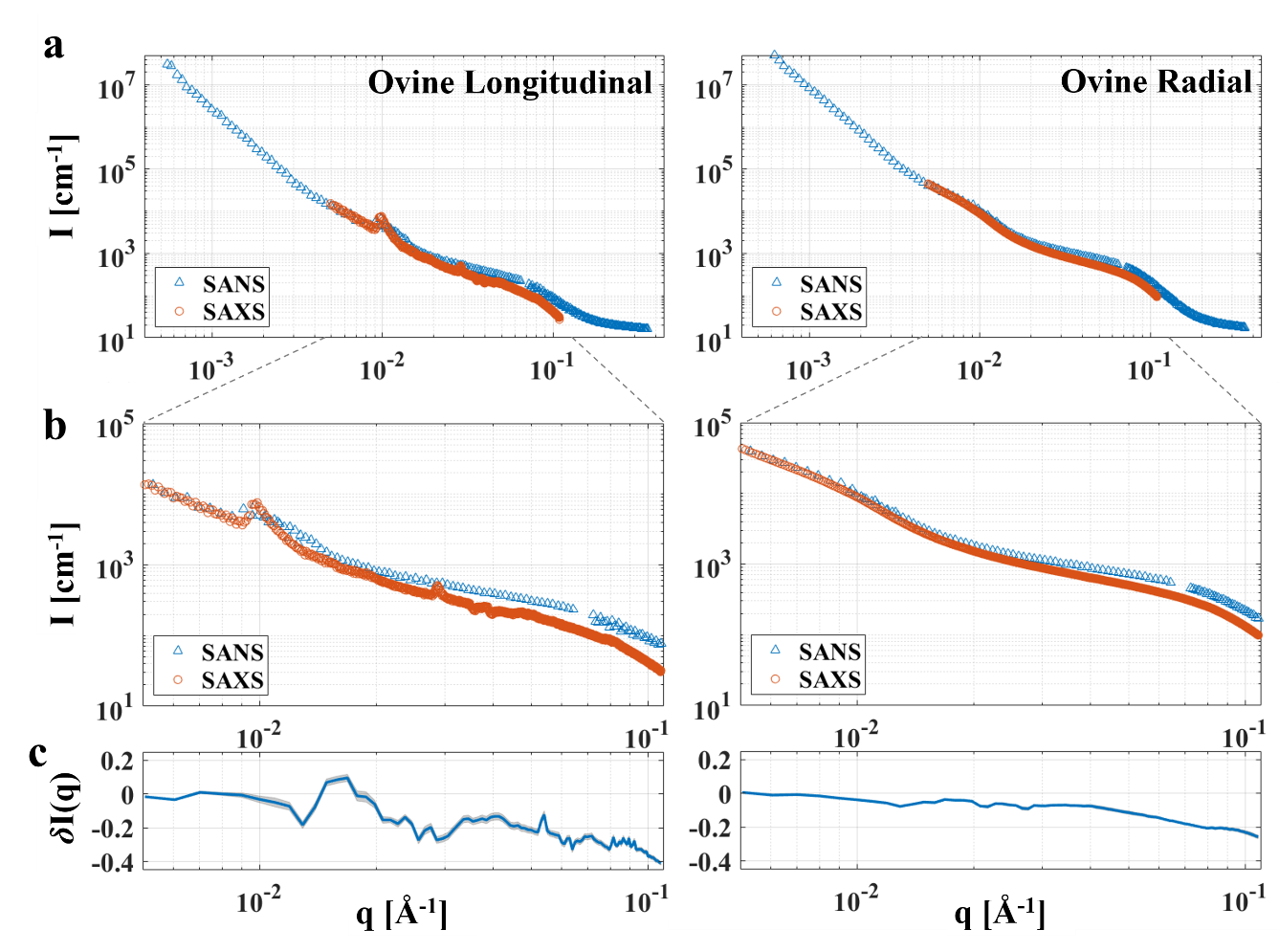


**Supplementary Figure 3.**  **a**) Full 2π integration (360°, log-log scale) of SANS (∆) and SAXS (o) patterns for the **ovine** longitudinal (left) and radial (right) specimens. The SAXS data was offset to overlap with the SANS data at low q. **b**) Zoom-in on the overlapping q-range for the plots in **a**). **c**) q-dependent intensity differences (linear-log scale), shown as mean (blue line) and standard deviation (grey shaded area) for the three measurement points. The figure was created using Matlab (R2019a).


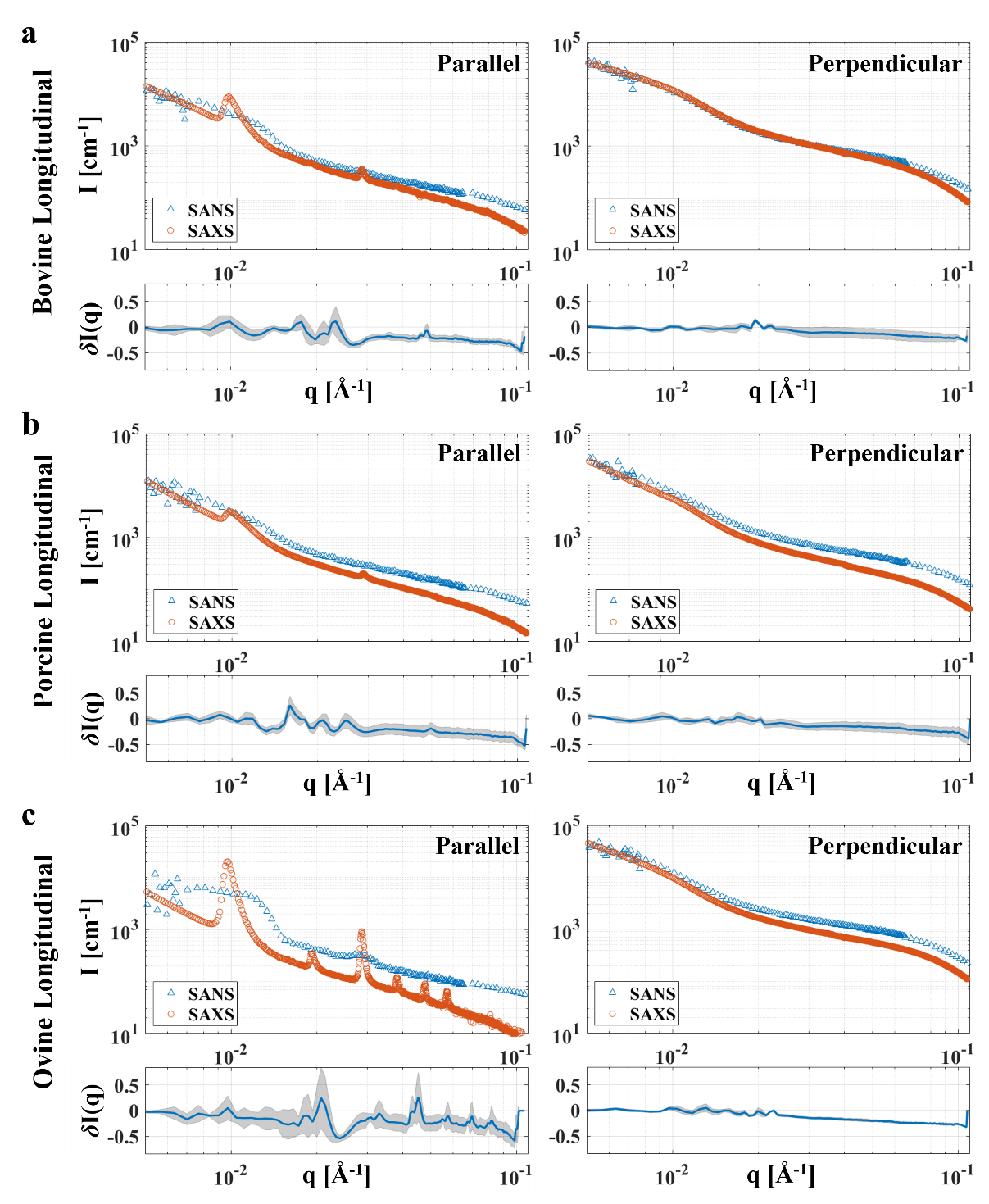


**Supplementary Figure 4**. Partial integration (parallel and perpendicular to the collagen fibre orientation, log-log scale) of SANS (∆) and SAXS (o) intensities in the overlapping q-range, for the **longitudinal specimens,** with q-dependent intensity differences (linear-log scale), shown as mean (blue line) and standard deviation (grey shaded area) for the three measurement points on each specimen. The SAXS data was offset to overlap with the SANS data at low q. **a**) Bovine specimens. **b**) Porcine specimens. **c**) Ovine specimens, for which the SAXS data showed very clear collagen peaks when integration was done parallel with the collagen fibre orientation. The figure was created using Matlab (R2019a).


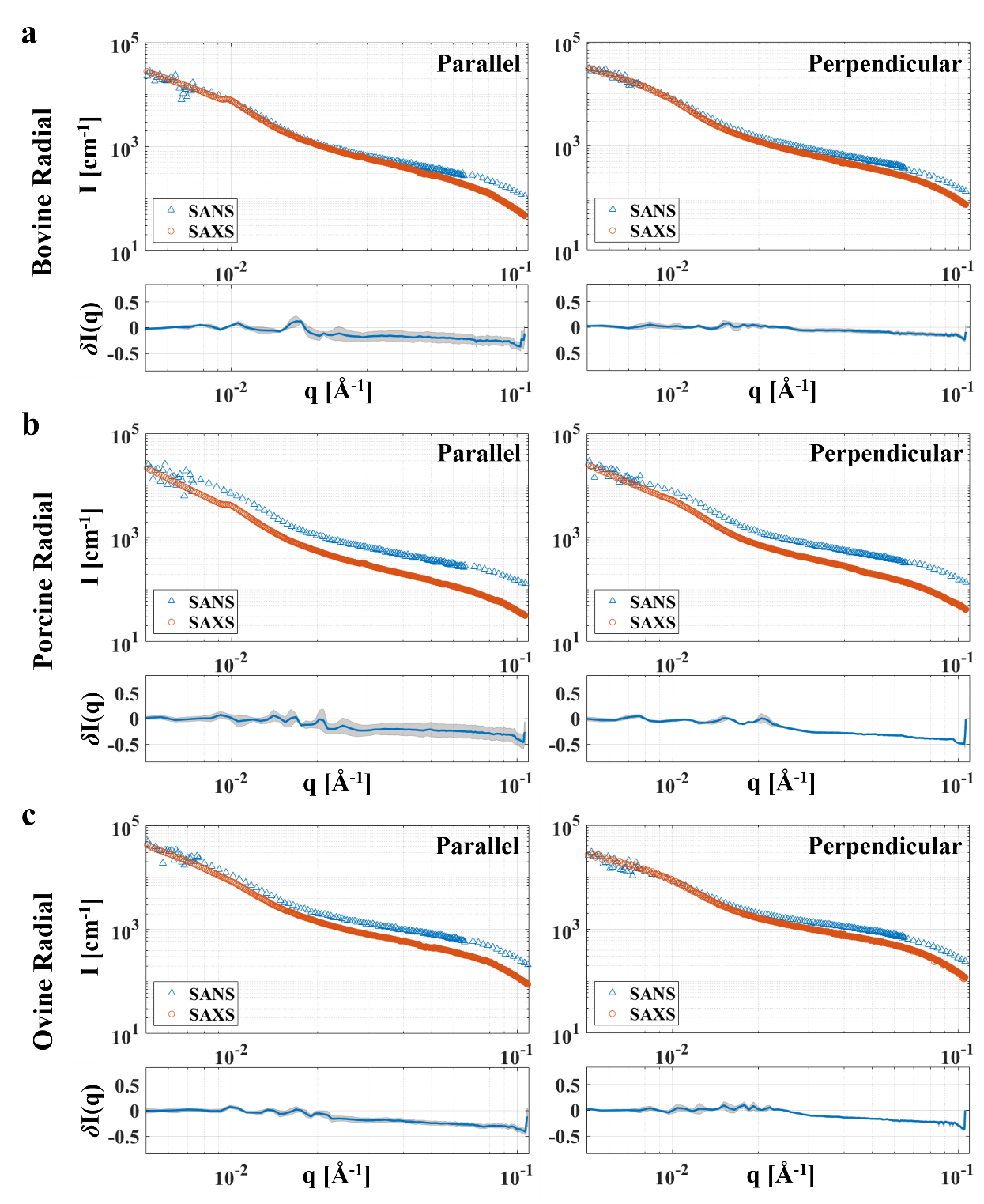


**Supplementary Figure 5**. Partial integration (parallel and perpendicular to the collagen fibre orientation, log-log scale) of SANS (∆) and SAXS (o) intensities in the overlapping q-range, for the **radial specimens,** with q-dependent intensity differences (linear-log scale, shown as mean (blue line) and standard deviation (grey shaded area) for the three measurement points on each specimen. The SAXS data was offset to overlap with the SANS data at low q. **a**) Bovine specimens. **b**) Porcine specimens. **c**) Ovine specimens. The figure was created using Matlab (R2019a).


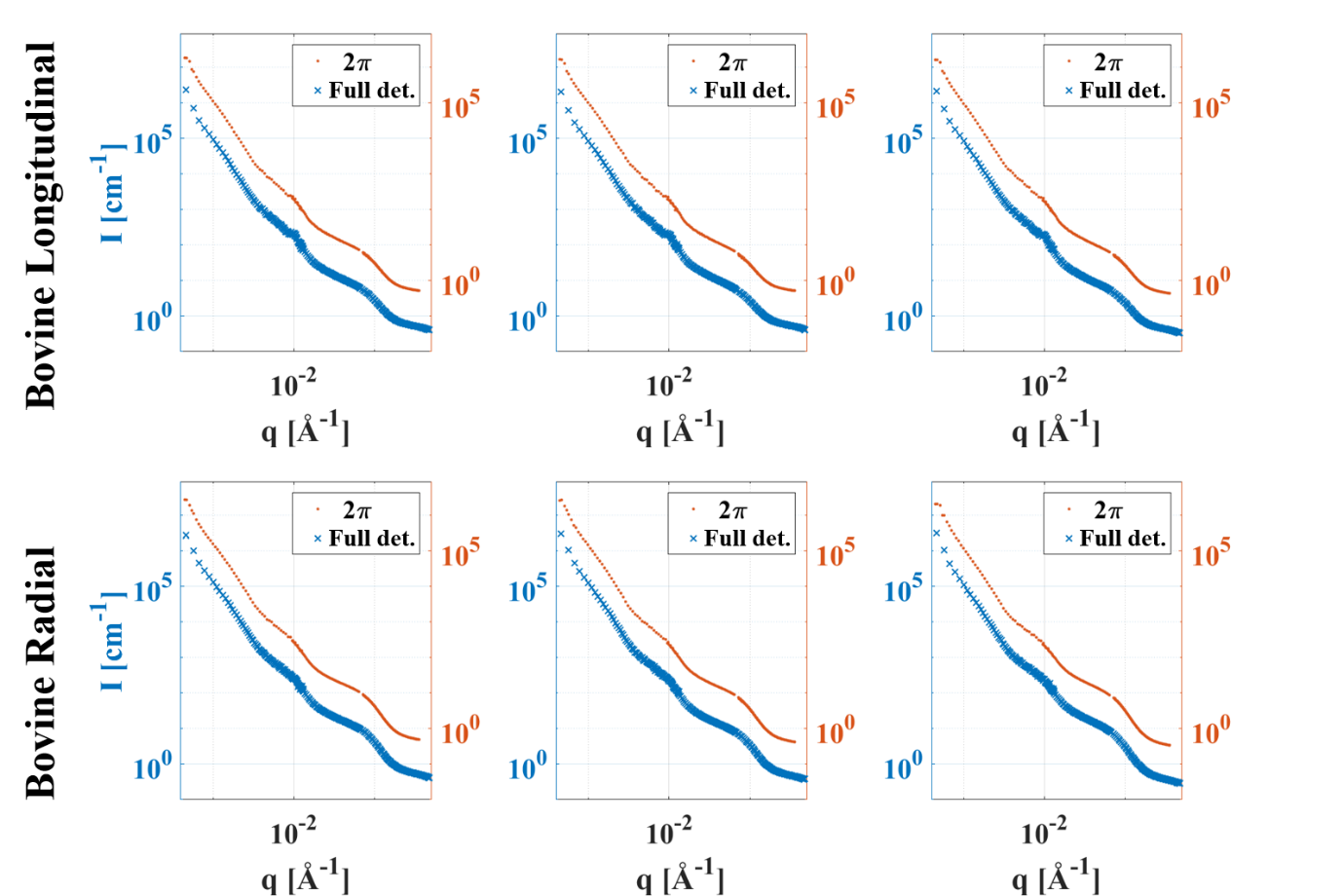


**Supplementary Figure 6**. SANS data from the all measurement positions on the longitudinal and radial **bovine** **specimens**, full integration (360°, log-log scale) over 2π (red dots, right y-axis) and the full detector (blue crosses, left y-axis, including detector bank corners) for all three sample-detector distances (full q-range). The figure was created using Matlab (R2019a).


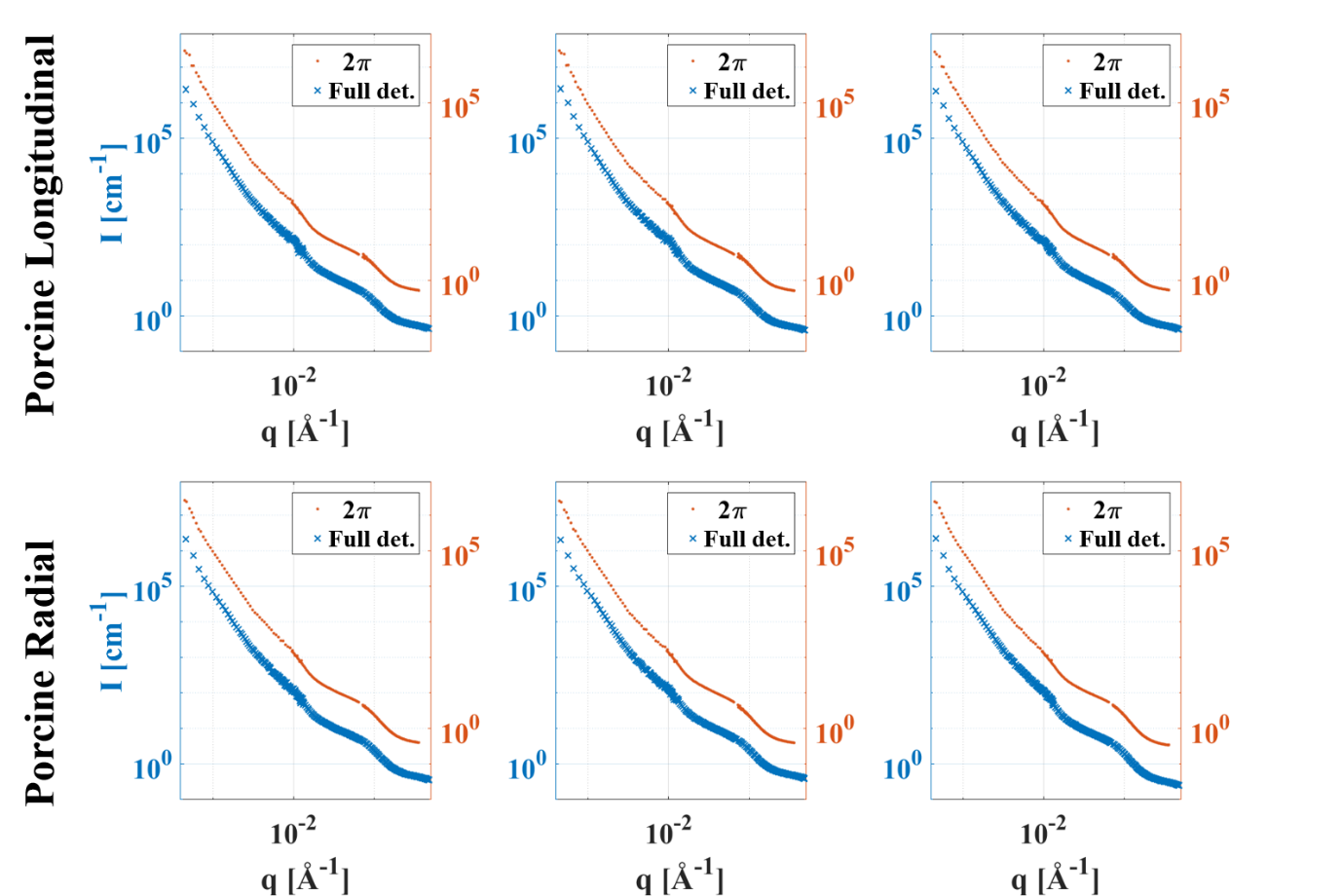


**Supplementary Figure 7**. SANS data from the all measurement positions on the longitudinal and radial **porcine specimens**, full integration (360°, log-log scale) over 2π (red dots, right y-axis) and the full detector (blue crosses, left y-axis, including detector bank corners) for all three sample-detector distances (full q-range). The figure was created using Matlab (R2019a).


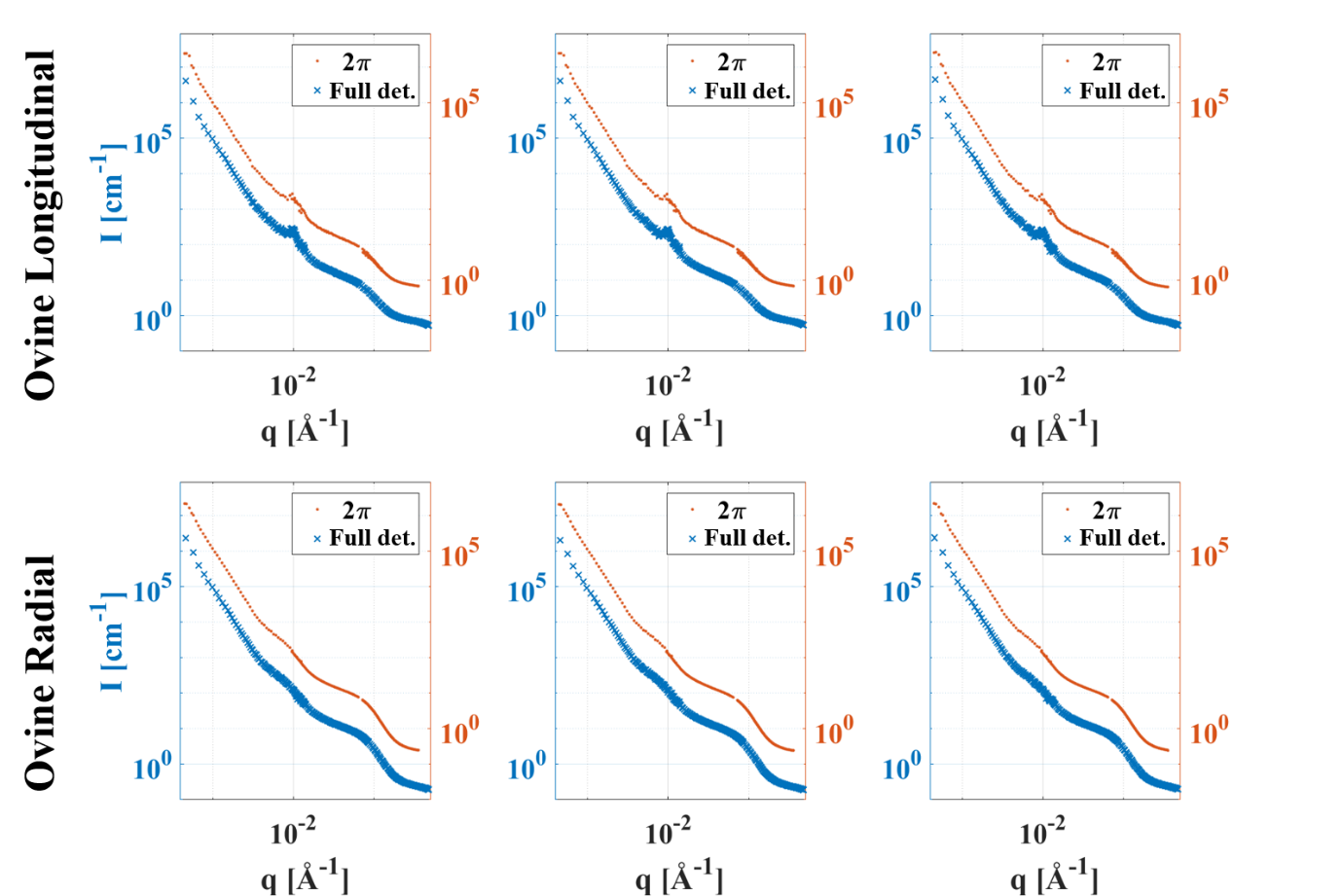


**Supplementary Figure 8**. SANS data from the all measurement positions on the longitudinal and radial **ovine specimens**, full integration (360°, log-log scale) over 2π (red dots, right y-axis) and the full detector (blue crosses, left y-axis, including detector bank corners) for all three sample-detector distances (full q-range). The figure was created using Matlab (R2019a).


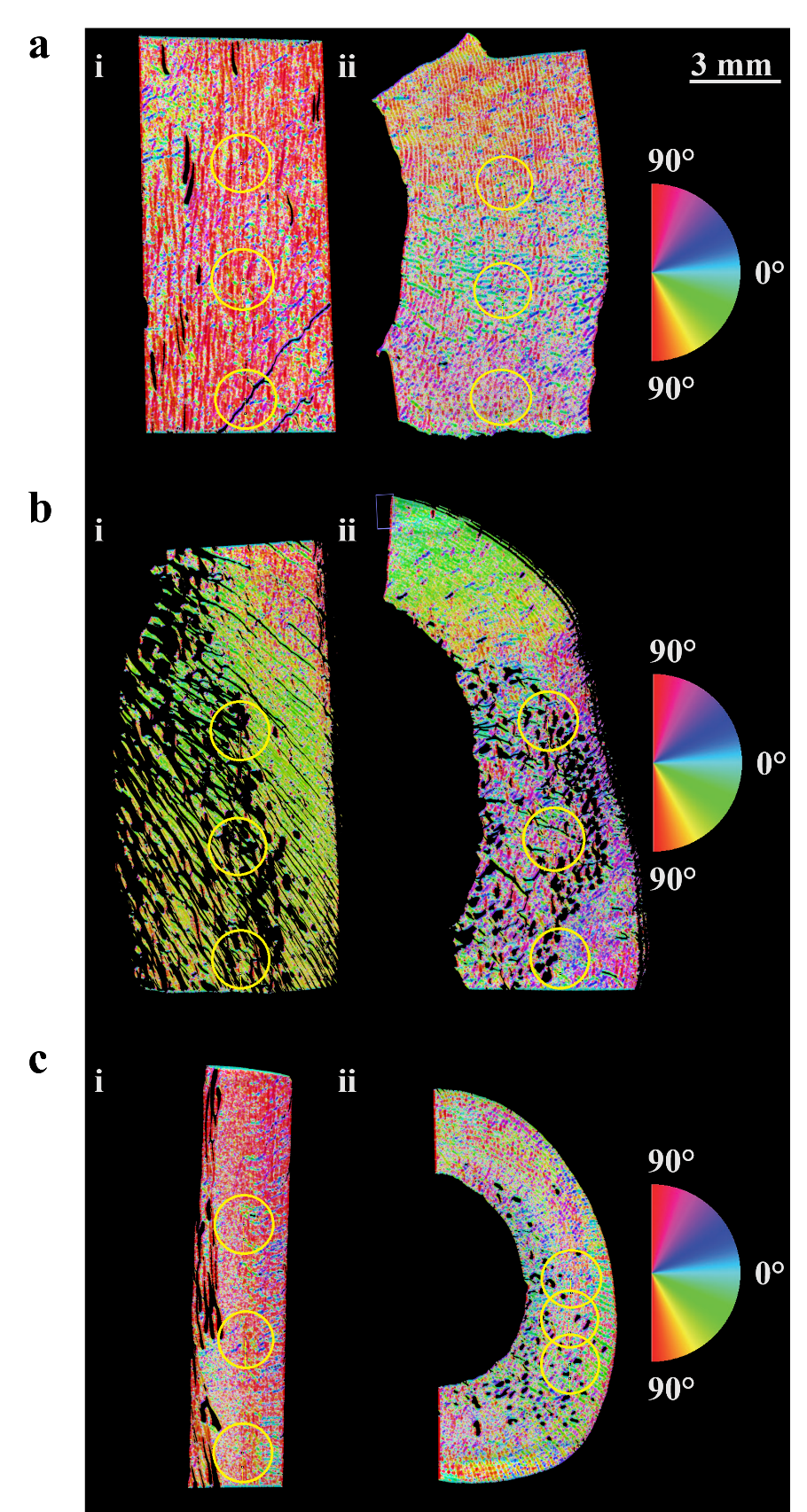


**Supplementary Figure 9**. Microstructural orientation obtained from micro-CT images, with measurement positions indicated with yellow circles. **a**) Longitudinal (i) and radial (ii) bovine specimen. **b**) Longitudinal (i) and radial (ii) porcine specimen. **c**) Longitudinal (i) and radial (ii) ovine specimen. The figure was created using ImageJ (v1.52i).
